# Supplementary material for: A high-entropy manganite in an ordered nanocomposite for long-term application in solid oxide cells
Source: Nat Commun. 2021 May 11;12:2660. doi: 10.1038/s41467-021-22916-4 (PMC8113253; doi:10.1038/s41467-021-22916-4)
Supplement: Supplementary file 1 — Supplementary Information [file 41467_2021_22916_MOESM1_ESM.pdf]

Supplementary Information

A high-entropy manganite in an ordered nanocomposite for long-term application in solid oxide cells

F. Baiutti (1), F. Chiabrera (1), M. Acosta (2), D. Diercks (3), D. Parfitt (4), J. Santiso (5), X. Wang (6), A. Cavallaro (7), A. Morata (1), H. Wang (6), A. Chroneos (4), J. MacManus-Driscoll (2), A. Tarancon (1,8)

(1) Catalonia Institute for Energy Research (IREC), Jardins de Les Dones de Negre 1, 08930 Sant Adrià del Besòs, Barcelona, Spain
(2) Department of Materials Science and Metallurgy, University of Cambridge, 27 Charles Babbage Road, Cambridge CB3 0FS, United Kingdom
(3) Department of Metallurgical and Materials Engineering, Colorado School of Mines, Golden, CO 80401, United States
(4) Faculty of Engineering, Environment and Computing, Coventry University, Priory Street, Coventry CV1 5FB, United Kingdom
(5) Catalan Institute of Nanoscience and Nanotechnology, ICN2, CSIC and The Barcelona Institute of Science and Technology (BIST), Campus UAB, 08193 Bellaterra, Barcelona.
(6) School of Materials Engineering, Purdue University, West Lafayette, Indiana 47907, United States
(7) Department of Materials, Imperial College London, Prince Consort Road, London SW7 2BP, United Kingdom
(8) ICREA, 23 Passeig Lluís Companys, Barcelona 08010, Spain.

Supplementary Notes

1. Structural characterization of LSM-SDC nanocomposites.

Supplementary Figure 1 depicts the different XRD measurements performed in the films. The standard 2θ/ω scan, in Supplementary Fig. 1a, gives information about the different crystallographic planes oriented parallel to the sample surface. The main contribution to the pattern corresponds to the 00L reflections of the YSZ substrate. At lower angles from the substrate peaks there are clear peaks coming from the film which correspond to 00L oriented SDC fluorite. The same peaks have been labelled as HH0 LSM which would correspond to the perovskite phase with a perfect vertical alignment having 110-orientation parallel to 001 of the SDC fluorite (LSM is indexed in a primitive pseudo-cubic perovskite cell for simplicity) structure. Please note that the very close bulk lattice spacing between LSM (110) (d =5.524 Å – cf. ICSD 152680) and SDC (001) (d =5.433 Å – cf. ICSD 01-075-0158) allows full out-of-plane coherence between the two structures. In the same scan there is another set of peaks indexed as 00L LSM', which corresponds to a residual part of c-axis oriented perovskite phase. The (') symbol is shown to differentiate from the 110-oriented LSM phase. In order to ascertain the in-plane arrangement, ϕ-scans were performed on asymmetric reflections of the different phases of the film with fixed 2θ and χ angles, as shown in Supplementary Fig.

1b. YSZ 202 ϕ-scan reflections (2θ=50.08º; χ=45.0º) show very narrow peaks with four-fold symmetry (90º apart in ϕ) corresponding to the cubic fluorite structure. The peaks indicate the [100]/[010] in-plane crystallographic directions of the substrate. The SDC 111 reflection ϕ-scan (2θ=28.12º; χ=53.7º) shows also four peaks at 45º from previous [100]/[010] directions. No additional components of the SDC are observed in this ϕ-scan. This is consistent with cube-on-cube epitaxy of SDC/YSZ with both having fluorite structure (despite the large mismatch between equilibrium cell parameters). The SDC 202 ϕ-scan (2θ=46.85º; χ=45.0º) shows the expected peaks at 0º as in the YSZ 202 case. However, it shows four additional peaks at 45º in ϕ indicated with an asterisk (*). Those peaks do not correspond to any other SDC orientation. Therefore, they should correspond to 002 peaks of a close matching LSM structure with a 110 orientation, which overlaps with the SDC 202 peaks in the 2θ and χ angles. For a clearer analysis of LSM orientation the corresponding 111 LSM reflections for the 110-oriented domains were measured (2θ=40.02º; χ=35.2º). At these angles there is no possible overlap with any of peak from the SDC or YSZ structures. The scan shows four main components at 0º, which would correspond to the LSM relative orientation depicted in Supplementary Fig. 1c. For this specific LSM orientation, the corresponding 111 reflections would have two-fold symmetry, with peaks at 0 and 180º. The presence of four peaks is an indication of the formation of two variants one rotated 90º in-plane with respect to the other. Additionally, the ϕ-scan shows another four components at 45º (indicated with *), which correspond to a minor variant of 110-oriented LSM rotated in-plane by 45º with respect to the main components (not depicted in scheme in Fig. 1d). These variants are also responsible for the previous secondary peaks in the SDC 202 ϕ-scan. The reciprocal space map in main text, Fig. 1e is a combination of measurements around different positions at the same ϕ angle. The cut passes by the 404 and 204 YSZ substrate reflections. In close proximity to the substrate peaks lay the corresponding 404 and 204 SDC reflections. The 110-oriented LSM components show in this cut the 222 and 221 reflections, which are clearly resolved from SDC. In case there is a close matching 90º-rotated LSM variant, as demonstrated in the ϕ-scans, the corresponding 400 and 310 LSM peaks are fully overlapped with those of SDC and cell parameters cannot be distinguished. The other LSM variants 110-oriented LSM rotated 45º in-plane and 001-oriented LSM do not contribute to this RSM cut.

2. Atom Probe Tomography

The background-subtracted compositions within the phases as determined by APT are shown in Table 1, main text. Differences in evaporation fields of the two different phases may lead to ion trajectory aberrations resulting in less-sharp interfaces and apparent intermixing between the phases in the reconstructions that was not actual present in the original material.[1,2] While this would seem to account for some of the observed intermixing, this effect would be observed most strongly in the 1 – 2 nm nearest the phase boundaries and would be less-pronounced or non-existent in the middle of the phases. As can be seen in main text Fig. 2c, the observed intermixing of cations is generally continuous throughout the whole region of each phase. Additionally, trajectory aberrations would affect all cations from a given phase, whereas the observed intermixing was more prevalent for some cations than others.

The field conditions present during analysis by APT have been shown to affect the measured stoichiometry of ceramics and oxides in particular.[3–5] The loss of oxygen due to neutral species formed during molecular dissociation appears to be the greatest contributor to this effect.[6,7] Therefore, prior to the APT analysis of the LSM-SDC VAN structure, APT analysis under a range of conditions were explored for LSM and SDC

individually. Results are reported in Supplementary Table 1. From these it was found that the applied fields producing values closest to the nominal stoichiometries occurred over a laser energy range of ~0.5 – 25 pJ. It is noted that under all of these analysis conditions, the total measured oxygen concentration in these individual phases was still ~ 3 – 12 at% below their nominal values. This is also observed in the oxygen concentrations measured in the mixed phase VAN materials (eg. main text, Table 1), but is not expected to have any effect on the analysis of the ^{18}O concentration profile (which is based on the ratio $O^{18}/(O^{18}+ O^{16})$ – see Supplementary Note 3) . The reduced ^{18}O concentration at the very top of the tip (≈10-15 nm), which can be observed in main text Fig. 2d and in Supplementary Figure 4 can be ascribed to a certain degree of oxygen back-exchange possibly occurring during or after focused ion beam preparation of the specimen.

3. FEM simulations

Finite Element Method (FEM) was employed to simulate the oxygen distribution in the VAN thin films after the O^{18} exchange measured by APT. FEM simulations were performed by COMSOL Multiphysics using the *Transport of diluted species* module. A 3D geometry consisting of LSM columns embedded in an SDC matrix was considered for taking in account the heterogeneous oxygen diffusion inside the VAN thin films (see Supplementary Figures 5a and b). The thickness of the layer was set to 60 nm according to the TEM measurements. The diameter of the LSM column was optimized to reproduce the cationic distribution in the APT tips (Mn and Ce maps of Fig. 2 in the main text and Supplementary Figure 4, the latter geometry not shown here). The time-dependent diffusion equation was solved for all solid domains, considering two different diffusion coefficients for LSM and SDC regions (D^*_{LSM} and D^*_{SDC} in Supplementary Figure 5). Since the diffusivity of SDC is orders of magnitude higher than LSM, a fixed value of $D^*_{SDC} = 1 \cdot 10^{-11}$ $cm^2 \cdot s^{-1}$ was considered. The adopted boundary conditions are sketched in the cross section view of Supplementary Supplementary Figure 5. On the top surface, a convection-type equation was set while all other surfaces were considered as adiabatic (zero oxygen flux). Since the quantification of the oxygen distribution by APT is limited to the lower part of the film (cf. main text and Supplementary Note 2), a single homogenous oxygen surface incorporation parameter k* was adopted. This hypothesis is valid since, in such regions and owing to the very high height on diameter dimensional ratio, oxygen diffusion in LSM columns is expected to take place laterally from the SDC matrix (see main text for the discussion about the mechanism of oxygen incorporation). Moreover, this way the oxygen surface incorporation parameter k* of the simulations can be directly compared to the averaged parameter extracted from the electrochemical measurements as, due to the high diffusivity of the SDC, the relative O^{18} concentration in the ceria matrix will be defined by the total amount of oxygen entering from the surface, i.e. from the averaged k* parameter. The simulations were computed in time dependent mode, setting the solution time equal to the exchange time of the isotope exchange experiments (6000 s) and the atmosphere $O^{18}/(O^{18}+ O^{16})$ equal to 0.9 (cf. main text methods). Finally, free tetrahedral elements were used for the meshing of the geometry, with a mesh refinement inside the LSM columns for better catching lateral diffusion profiles.

A parametric sweep of D^*_{LSM} and k* was simulated in order to achieve a good fitting of the $O^{18}/(O^{18}+ O^{16})$ profiles extracted by APT (Figure 2 in the main text and Supplementary Figure 4). Supplementary Figure 6(a) to (c) show the spatial dimensional maps and cross sections of relative $O^{18}/(O^{18}+ O^{16})$ obtained for the best fitted parameters ($D^*_{LSM} = 3.6 \cdot 10^{-17}$ $cm^2 \cdot s^{-1}$ and $k^* = 2.4 \cdot 10^{-9}$ $cm \cdot s^{-1}$). The relative oxygen

concentration is observed to be constant in the SDC matrix and to be gradually diffusing into the LSM columns, generating a lateral concentration gradient. The surface of LSM is characterized by a high concentration of $O^{18}/(O^{18}+O^{16})$, due to the sluggish diffusivity in this material that leads to an accumulation of O^{18} in the subsurface region. It must be noticed that, if an heterogenous oxygen incorporation process was considered, the subsurface region would drastically vary its concentration profile. Nevertheless, these effects would not affect the lower part of the thin film, where the diffusion in the LSM takes place mainly from the homogenous SDC matrix, as described above.

A comparison with the experimental data extracted from the APT shows that the model is able to successfully reproduce the modulation of concentration passing from the SDC matrix to the LSM columns, due to the lower diffusivity of LSM (see Figure 2e of the main text and Supplementary Figure 4b). The values of D_{LSM}^{*} and k* extracted from the simulation are in fair agreement with the diffusion in LSM thin films and with the surface exchange coefficient obtained by EIS (cf. Supplementary 7 and main text Figure 3c, respectively), supporting the validity of the method for measuring oxygen diffusion in heterogenous media at the nanoscale.

4. Impedance spectroscopy

The equivalent circuits used for the fitting of the LSM-SDC and for the LSM impedance arcs are reported in Supplementary Figure 7, panels (a) and (b), respectively. For LSM-SDC, the physically meaningful equivalent circuit of choice is typically employed in the case of surface-limited cathodic processes.[8,9] Here R_s (R_{Ag}),C_s (C_{Ag}) represent the polarization (Ag counter-electrode) resistance and parallel area-specific-capacitance, respectively; R_{YSZ} is the serial resistance, mainly determined by the YSZ substrate. Capacitances C are modeled as constant phase elements CPE having impedance $\boldsymbol{Z_{CPE}} = \frac{\mathbf{1}}{[\boldsymbol{T}(\boldsymbol{i\omega})^{\boldsymbol{P}}]}$, $\boldsymbol{\omega}$ being the angular frequency of the AC signal, T and P being the fitting parameters. From here, the true capacitance is calculated as $\boldsymbol{C} = (\boldsymbol{R}^{\mathbf{1}-\boldsymbol{P}}\boldsymbol{T})^{\frac{\mathbf{1}}{\boldsymbol{P}}}$. Contributions stemming from the mass transport in the electrode are negligible. For LSM, the equivalent circuit of choice, initially derived by J.Jamnik et al.,[10] is typically used as a physically meaningful circuit for the analysis of mixed ionic-electronic conductors.[11–14] Here C_{chem}: area-specific chemical capacitance; R_i: resistance to transport of oxygen ions in the bulk of the film (the electronic resistance is negligible); R_s,C_s :surface resistance and area-specific-capacitance for the oxygen chemical reaction, respectively; C_e: capacitance describing the electron blocking surface at the electrode-electrolyte interface; R_{YSZ} :serial resistance determined by the YSZ substrate. C_s is modeled as a constant phase element. The impedance contribution of the Ag counter-electrode is here negligible and has not been taken into consideration for the fittings. The results from the fittings are reported in Supplementary

Supplementary Table *2* (LSM-SDC) and Supplementary Supplementary Table 3 (LSM). From here, the oxygen kinetics parameters were extracted, i.e. surface exchange coefficient $\boldsymbol{k^q} = \boldsymbol{k_b \cdot T} \big/ \boldsymbol{R_s \cdot c_{O_2} \cdot z_i^2 \cdot e^2}$ (values reported in Fig. 3, main text) and oxygen diffusion coefficient $\boldsymbol{D^q} = \boldsymbol{k_b \cdot T} \big/ \boldsymbol{R_i \cdot c_{O_2} \cdot z_i^2 \cdot e^2}$ (Supplementary Figure 8), $\boldsymbol{c_{O_2}}$ being the concentration of oxygen in the lattice and $\boldsymbol{z_i}$ the number of charges involved in the transport and oxygen diffusion coefficient. The area-specific-resistance for LSM and LSM-SDC, reported in Supplementary Figure 9, is calculated as ASR= R_i+ R_s (R_i is negligible for LSM-SDC). In Supplementary Figure 11, a graphical representation of the fitting results for the pO_2-dependendent electrochemical behavior for LSM are shown. The data related to LSM-SDC are reported in the main text, Figure 3 (in the form of $\boldsymbol{k^q}$ vs pO_2).

5. DFT and Bond Valence

Energy minimised structures from the DFT calculations were used to examine the effects of strain and dopants on the relaxed internal atomic coordinates. Various different configurations of cations were examined along with differing levels of isostatic strain imposed upon the simulation cell. As with previous theoretical results on defect properties of LSM and related perovskites, we found little influence of specific cation ordering on the energy of the cell. This is also in agreement with the observed lack of static ordering in the reported LSM diffraction data. Data are reported for a single cation configuration per composition and we believe this to be representative of the configurational average.

For each of the different cation distributions we quantified the stability of the cations relative to the surface of the cell we consider the bond valence sum at each lattice site. This is given by:

$$\text{Valence} = \sum_{i=1,N} exp\left(\frac{R_0 - R}{b}\right)$$

Where R_0 and b are empirical parameters, R is the distance between each cation and the ith neighbouring oxygen ion and the sum is taken over the N oxygen ions within 3.5Å, which in the perovskites structures studied, corresponds to 12 nearest oxygen ions (A-sites) and 8 nearest oxygen ions (Mn-site). Although the *A*-sites are not regular polyhedral, and in some descriptions are listed as between 9 and 12 fold coordinated, this is accounted for in the bond valence analysis by the reduction in valence sum contribution as the bond length increases. We summarise our bond valence parameters in Supplementary Table 4.

To provide additional crystallographic support to our bond valence calculations, we also provide the atomic volume, *V*, attributed to each cation lattice site. This is calculated according to the Voronoi cell constructed between each cation site and its 12 neighboring oxygen ions.

Supplementary Figures

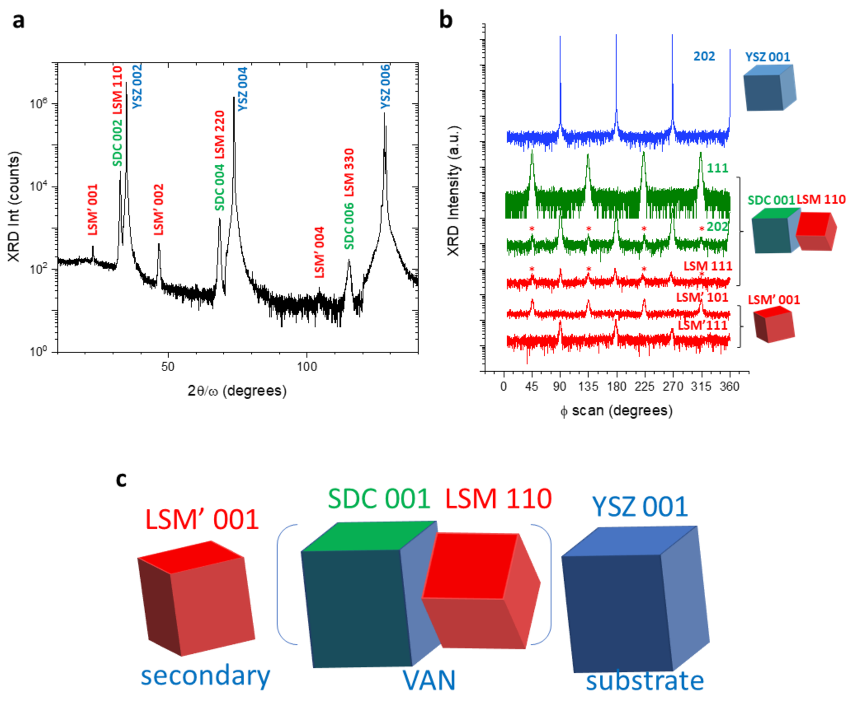

Supplementary Figure 1. XRD characterization of LSM-SDC nanocomposites. a) two theta-omega. b) phi scan. c) scheme of the observed orientations.

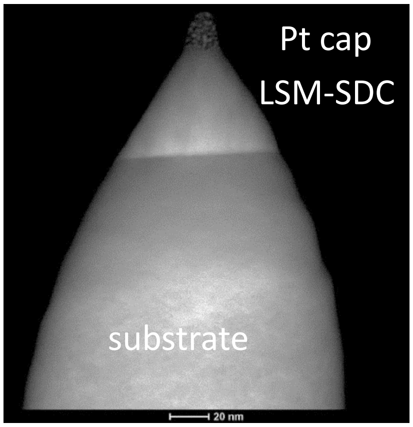

Supplementary Figure 2. HAADF-STEM image of a Ga^+ FIB-prepared APT specimen with the regions indicated within the image.

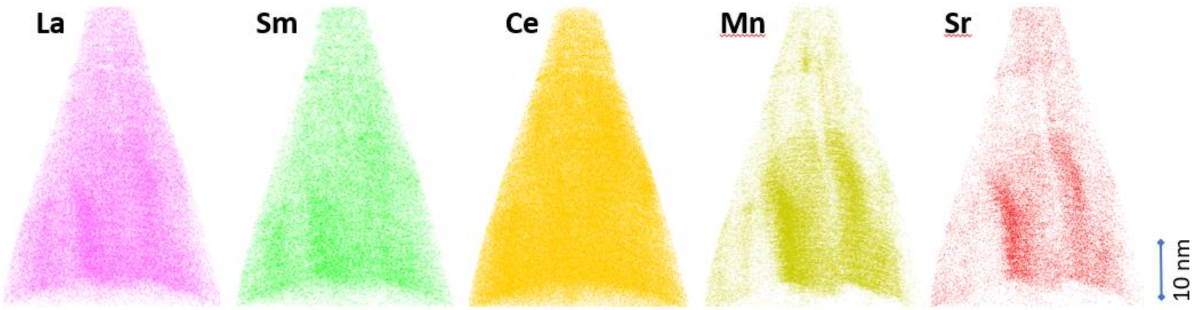

Supplementary Figure 3. Cation spatial distribution according to APT.

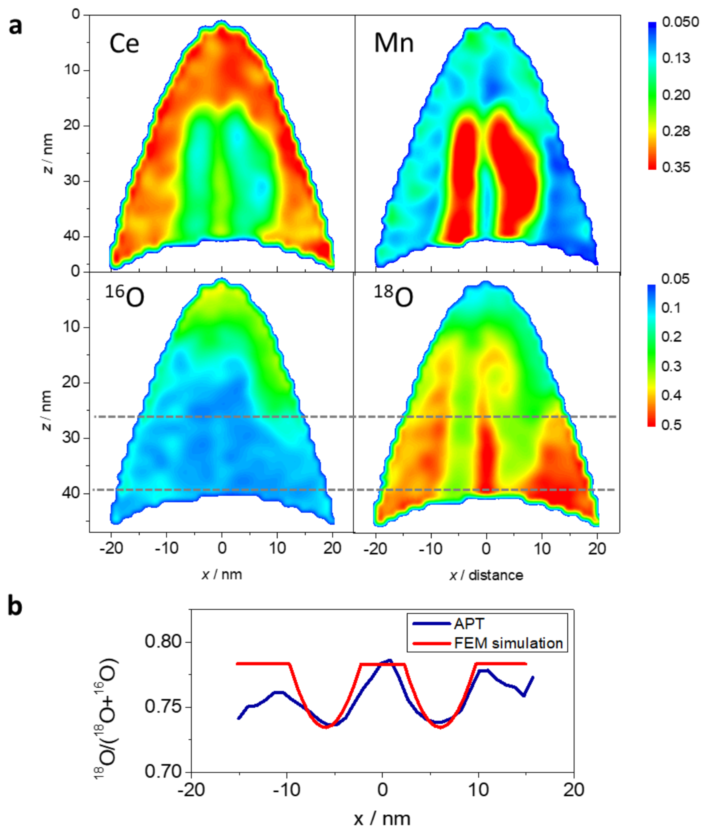

Supplementary Figure 4. a) Concentration colormaps for different elements from a 2D cross-section of the APT tip represented in main text, Figure 2. b) In blue, ^{18}O fraction profile, obtained integrating the ^{16}O and ^{18}O signals in the area between the dotted lines in panel (a). In red, result of profile fitting by FEM methods. The extracted oxygen surface exchange coefficient of the composite (k^q=2.4·10^{-9} cm·s^{-1}) and the diffusion coefficient of LSM (D^* =3.6·10^{-17} cm^2·s^{-1}) are reported in main text Fig. 3 and Supplementary Figure 8 for comparison.

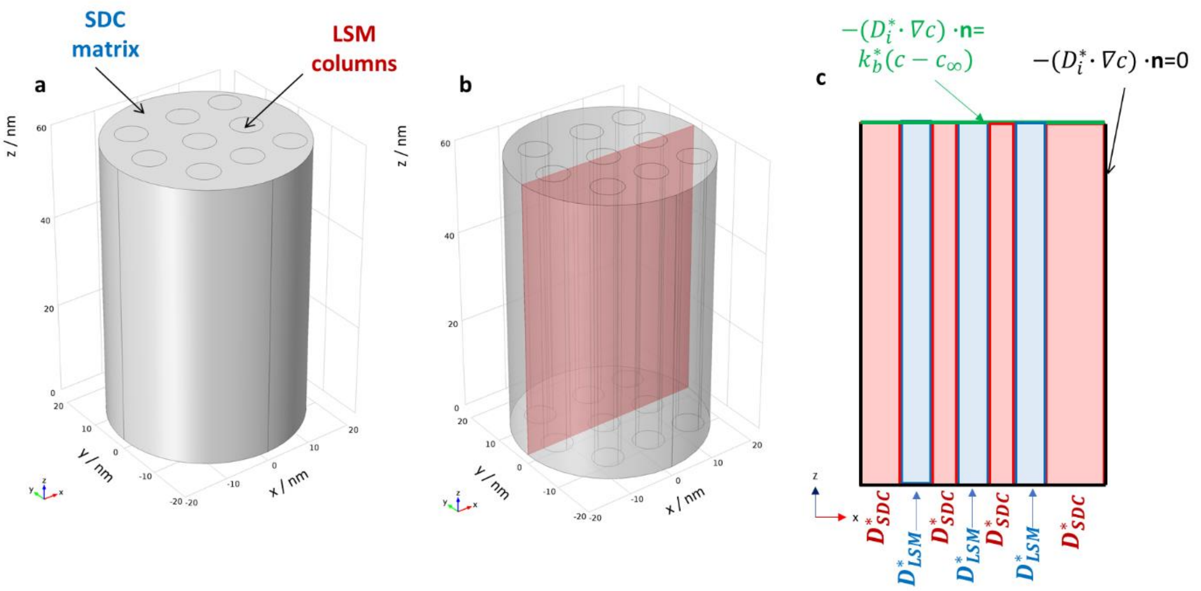

Supplementary Figure 5. a) and b) 3D geometry used in the FEM model for simulating the oxygen profiles extracted from APT. c) Sketch of the cross-sectional area drawn in red in b) showing the main boundary conditions adopted and the different diffusivity parameter set in the SDC matrix and in the LSM columns.

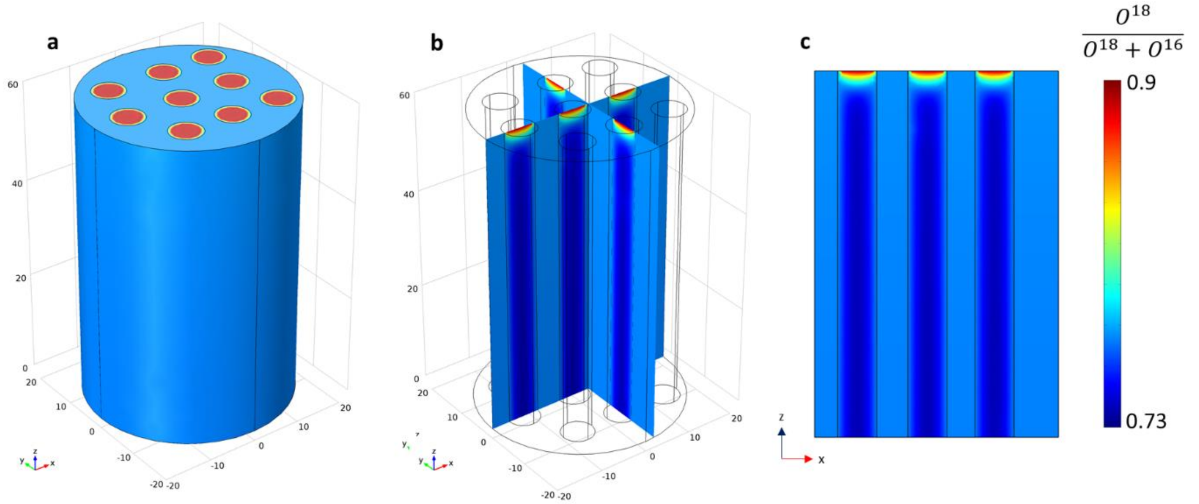

Supplementary Figure 6. a) 3D map of the relative O^{18} concentration simulated for $\boldsymbol{D^*_{LSM} = 3.6 \cdot 10^{-17}}$ $cm^2 \cdot s^{-1}$ and $\boldsymbol{k^* = 2.4 \cdot 10^{-9}}$ $cm \cdot s^{-1}$ after 6000 s. b) and c) Cross sectional planes showing the constant O^{18} concentration in the SDC matrix and the concentration profiles in the LSM columns.

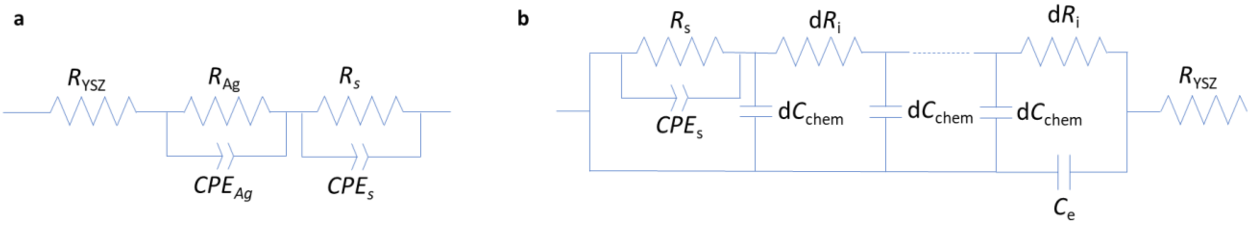

Supplementary Figure 7. Equivalent circuits used for the fitting of the LSM-SDC (a) and LSM (b) impedance data.

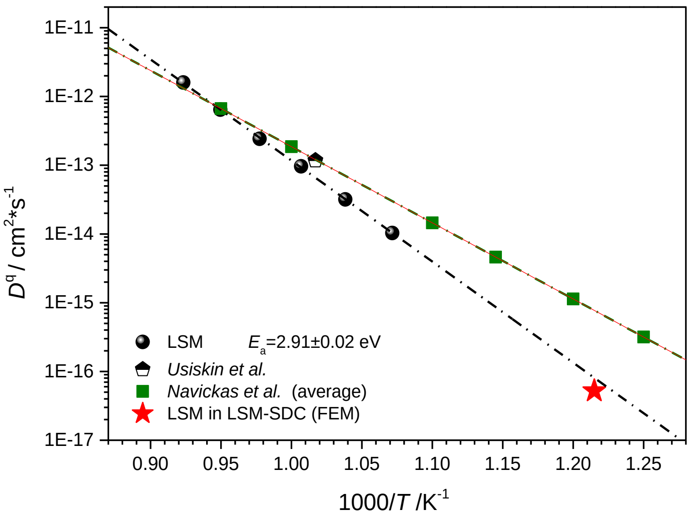

Supplementary Figure 8. Oxygen diffusion coefficient D^q for LSM derived from EIS measurements (black points) and APT (FEM simulations – red star), compared to literature values (Usiskin et al. after Ref. 13, Navickas et al. after Ref. 15).

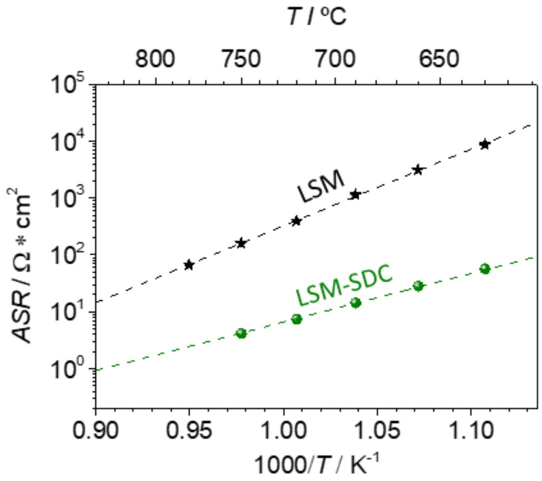

Supplementary Figure 9. Temperature dependence for the area-specific-resistance (ASR) in LSM and LSM-SDC.

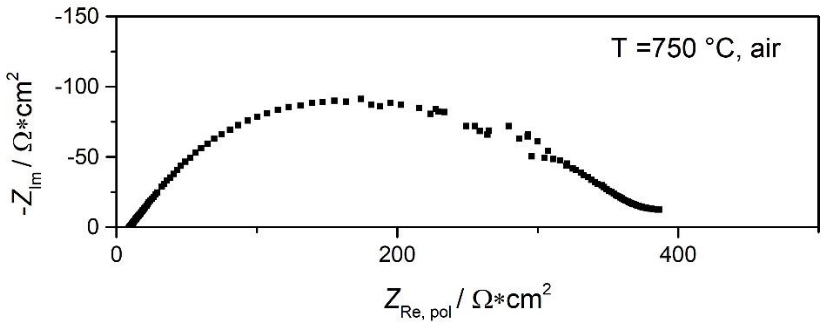

Supplementary Figure 10. Nyquist plot for symmetric SDC cells measured in air at 750 C.

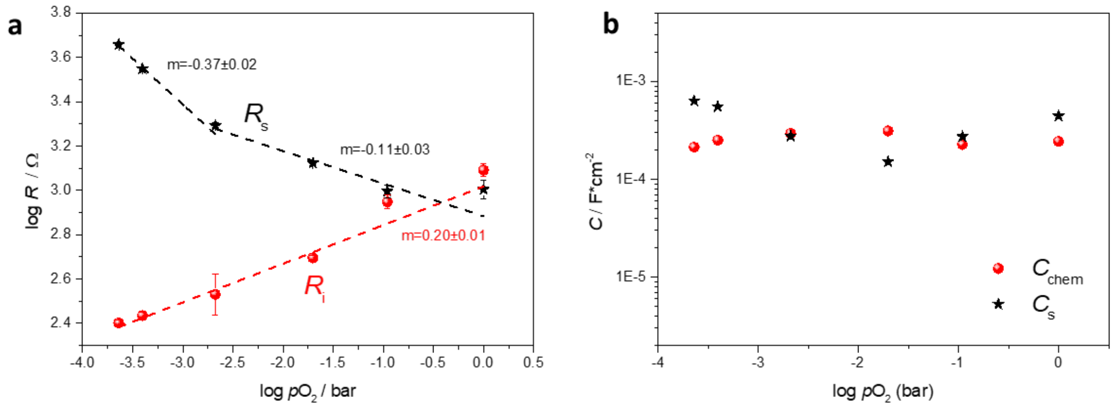

Supplementary Figure 11. Fitting results of EIS spectra for LSM as a function of pO_2. a) Surface resistance (R_s) and oxygen diffusion resistance (R_i). b) Area-specific capacitance for the oxygen incorporation process (C_s) and chemical capacitance (C_{chem}). Here, the error bars are smaller than the symbols.

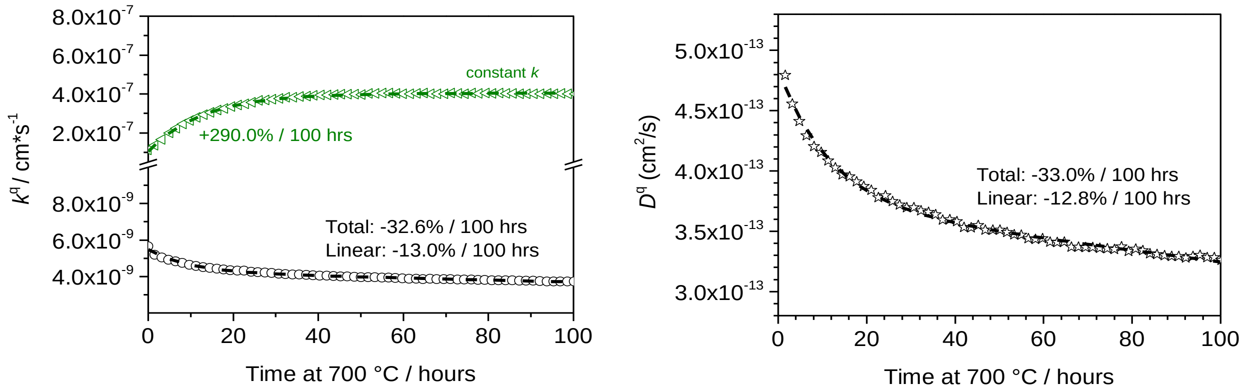

Supplementary Figure 12. Oxygen kinetics parameters retrieved from EIS during thermal ageing test for LSM (black) and SDC-LSM (green). a) Surface exchange coefficient. b) Diffusivity. A qualitative fitting of the experimental values has been obtained by considering a linear and an exponential component ($y=y_0+mx+A*exp(-x/t)$).

Supplementary Tables

Supplementary Table 1. Measured at.% by APT on LSM and SDC single phase films for different laser energies. In green, the energy windows used for the final experiment on VANs are highlighted.

$La_{0.8}Sr_{0.2}MnO_3$ – measured at.%					
Laser (pJ)	La	Sr	Mn	O	RMS dev
0.5	0.17	0.05	0.23	0.54	0.07
1	0.18	0.05	0.23	0.53	0.08
2	0.19	0.05	0.24	0.52	0.10
4	0.20	0.05	0.24	0.50	0.11
7	0.21	0.06	0.25	0.49	0.13
10	0.22	0.06	0.24	0.48	0.14
14	0.22	0.05	0.24	0.49	0.13
18	0.21	0.05	0.23	0.50	0.12
24	0.19	0.04	0.23	0.54	0.08
30	0.16	0.01	0.15	0.68	0.10
nominal					
	0.16	0.04	0.2	0.6	

$Ce_{0.8}Sm_{0.2}O_{1.9}$ – measured at.%				
Laser (pJ)	Ce	Sm	O	RMS dev
0.8	0.29	0.08	0.63	0.03
1.5	0.29	0.08	0.63	0.03
3	0.29	0.08	0.63	0.03
6	0.30	0.08	0.62	0.04
12	0.30	0.08	0.62	0.05
18	0.31	0.09	0.61	0.06
24	0.31	0.09	0.60	0.06
30	0.31	0.09	0.60	0.07
nominal				
	0.28	0.07	0.66	

Supplementary Table 2. Resulting fitting parameters for LSM-SDC as a function of temperature.

T **(°C)**	$\boldsymbol{R_s}$ **(Ω*cm²)**	$\boldsymbol{C_s}$ **(F*cm⁻²)**	$\boldsymbol{T_s}$	$\boldsymbol{P_s}$	$\boldsymbol{R_{YSZ}}$ **(Ω*cm²)**	$\boldsymbol{R_{Ag}}$ **(Ω*cm²)**	$\boldsymbol{C_{Ag}}$ **(F*cm⁻²)**	$\boldsymbol{T_{Ag}}$	$\boldsymbol{P_{Ag}}$
750	4.14	9.82E-04	8.31E-04	0.95	2.54	0.26	6.14E-04	9.14E-04	0.64
720	7.39	9.78E-04	7.37E-04	0.97	3.36	0.38	8.93E-04	1.13E-03	0.61
690	14.34	9.46E-04	7.00E-04	0.97	4.67	0.56	1.27E-03	1.58E-03	0.56
660	28.33	9.18E-04	6.50E-04	0.97	6.76	0.78	9.75E-04	1.16E-03	0.57
630	56.66	9.01E-04	6.22E-04	0.97	10.04	1.21	1.02E-03	1.18E-03	0.54
600	118.02	8.91E-04	6.04E-04	0.97	15.28	1.99	1.18E-03	1.34E-03	0.51
570	242.05	8.96E-04	5.99E-04	0.97	25.02	2.56	7.15E-04	8.33E-04	0.53

Supplementary Table 3. Resulting fitting parameters for LSM as a function of temperature.

T **(°C)**	$\boldsymbol{C_{chem}}$ **(F*cm⁻²)**	$\boldsymbol{R_i}$ **(Ω*cm²)**	$\boldsymbol{R_s}$ **(Ω*cm²)**	$\boldsymbol{C_s}$ **(F*cm⁻²)**	$\boldsymbol{T_s}$	$\boldsymbol{P_s}$	$\boldsymbol{C_e}$ **(F*cm⁻²)**	$\boldsymbol{R_{YSZ}}$ **(Ω*cm²)**
810	2.66E-04	11.55	17.76	5.17E-04	2.72E-04	0.74	2.52E-05	1.46
780	2.76E-04	27.87	39.01	4.66E-04	2.59E-04	0.68	3.55E-05	1.73
750	2.41E-04	71.92	86.58	4.16E-04	1.88E-04	0.68	5.10E-05	2.11
720	1.88E-04	175.52	218.24	3.62E-04	1.33E-04	0.67	7.93E-05	3.10
690	4.58E-04	498.72	119.12	5.84E-03	8.27E-04	0.90	3.85E-04	4.49
660	1.80E-04	1534.08	1571.20	2.94E-04	6.17E-05	0.64	6.36E-05	6.72
630	7.55E-05	2180.16	6465.44	1.32E-04	2.23E-05	0.65	6.83E-05	11.10

Supplementary Table 4: Values of bond valence parameters used in the study

	**La**	**Mn**	**Sr**
r_0	2.086	1.74	2.118
b	0.45	0.417	0.37
**Formal valence**	+3	+3	+2
**Calculated valence sum in bulk LSM**	2.96	3.18	2.14

Supplementary References

1 M. K. Miller, *Le J. Phys. Colloq.*, 1987, **48**, C6-565-C6-570.

2 F. Vurpillot, A. Bostel and D. Blavette, *Appl. Phys. Lett.*, 2000, **76**, 3127–3129.

3 A. Devaraj, R. Colby, W. P. Hess, D. E. Perea and S. Thevuthasan, *J. Phys. Chem. Lett.*, 2013, **4**, 993–998.

4 R. Kirchhofer, M. C. Teague and B. P. Gorman, *J. Nucl. Mater.*, 2013, **436**, 23–28.

5 D. R. Diercks and B. P. Gorman, *Microsc. Microanal.*, 2016, **22**, 648–649.

6 B. Gault, D. W. Saxey, M. W. Ashton, S. B. Sinnott, A. N. Chiaramonti, M. P. Moody and D. K. Schreiber, *New J. Phys.*, 2016, **18**, 033031.

7 D. Zanuttini, I. Blum, L. Rigutti, F. Vurpillot, J. Douady, E. Jacquet, P. M. Anglade and B. Gervais, *Phys. Rev. A*, 20170 **95**, 061401.

8 S. B. Adler, *Chem. Rev.*, 2004, **104**, 4791–4843.

9 J. Januschewsky, M. Ahrens, A. Opitz, F. Kubel and J. Fleig, *Adv. Funct. Mater.*, 2009, **19**, 3151–3156.

10 J. Jamnik, *J. Electrochem. Soc.*, 1999, **146**, 4183.

11 J. Fleig, H. R. Kim, J. Jamnik and J. Maier, *Fuel Cells*, 2008, **8**, 330–337.

12 M. Kubicek, A. Limbeck, T. Frmling, H. Hutter and J. Fleig, *J. Electrochem. Soc.*, 2011, **158,** B727-B734.

13 R. E. Usiskin, S. Maruyama, C. J. Kucharczyk, I. Takeuchi and S. M. Haile, *J. Mater. Chem. A*, 2015, **3**, 19330–19345.

14 F. Chiabrera, A. Morata, M. Pacios and A. Tarancón, *Solid State Ionics*, 2017, **299**, 70–77.

15 E. Navickas, T. M. Huber, Y. Chen, W. Hetaba, G. Holzlechner, G. Rupp, M. Stöger-Pollach, G. Friedbacher, H. Hutter, B. Yildiz and J. Fleig, *Phys. Chem. Chem. Phys.*, 2015, **17**, 7659–7669.
